# PLAN standards and writing to patients: quality improvement by audit

**DOI:** 10.1192/bjo.2021.589

**Published:** 2021-06-18

**Authors:** Edie Shaw, Catherine Adams, Thomas Maclaren, Fergus Brown

**Affiliations:** 1 Foundation Doctor, Imperial College Healthcare NHS Trust; 2 Consultant Psychiatrist, Chelsea and Westminster Hospital; 3 Foundation Doctor, Chelsea and Westminster Hospital NHS Foundation Trust

## Abstract

**Aims:**

This quality improvement project aimed to assess the adherence of a hospital psychiatric liaison team's documentation of assessments to the Psychiatric Liaison Accreditation Network (PLAN) standards framework; to identify areas of improvement; to identify barriers to and improve adherence.

**Method:**

Data were extracted from 27 randomly selected patient assessments from 01/07/2020 to 31/08/2020 and then 27 assessments from 01/10/2020 to 30/11/2020 for re-audit.

Quantitative data was collected by calculating the percentage of assessments which documented each specific aspect of PLAN standards.

Qualitative data including attitudes specifically towards writing to patients was gathered from 1:1 discussions with members of staff.

Interventions between rounds of audit:

Presentation of results of 1st data collection to team in November 2020 followed by discussion

Emailed instructions to create a template based on PLAN standards for assessments to staff

Lobbied for Cerner access at liaison team office to facilitate use of above

**Result:**

Quantitative – overall improvements were seen in adherence to all aspects of documentation of assessments including collateral history (from 23% to 67%) past medical history (30% to 70%) and acknowledging the patient/carer perspective (46% to 74%). Some improvement was seen in offering written correspondence to patients (0% to 20%).

Qualitative – the majority of comments regarding writing to patients were positive, with no staff members opposing the standard (“it is best practice”, “should become a habit”). However, some barriers were identified including increased workload (“requires more editing”, “could take a lot more time”).

**Conclusion:**

Team adherence to PLAN standards for documentation of assessments was improved through low intensity interventions. Overall adherence was high, however certain areas leave space for improvement. The audit facilitated conversations around writing to patients on discharge, both in the form of formal gathering of qualitative data and informal discussions between staff. Attitudes towards writing these letters were positive and some improvement was seen between audits. Ongoing audit activity aims to further improve adherence and monitor improvements.

